# The risk of post-polio syndrome among immigrant groups in Sweden

**DOI:** 10.1038/s41598-023-33240-w

**Published:** 2023-04-13

**Authors:** Per Wändell, Kristian Borg, Xinjun Li, Axel C. Carlsson, Jan Sundquist, Kristina Sundquist

**Affiliations:** 1 Division of Family Medicine and Primary Care, NVS Department, Karolinska Institutet, Alfred Nobels Allé 23, 141 83 Huddinge, Sweden; 2 Center for Primary Health Care Research, Lund University, Malmö, Sweden; 3 Division of Rehabilitation Medicine, Department of Clinical Sciences, Karolinska Institutet, Danderyd University Hospital, Stockholm, Sweden; 4 Academic Primary Health Care Centre, Region Stockholm, Stockholm, Sweden; 5 Department of Family Medicine and Community Health, Department of Population Health Science and Policy, Icahn School of Medicine at Mount Sinai, New York, NY USA; 6 Department of Functional Pathology, Center for Community-Based Healthcare Research and Education (CoHRE), School of Medicine, Shimane University, Matsue, Japan

**Keywords:** Diseases, Medical research, Neurology

## Abstract

To examine the risk of post-polio syndrome (PPS) in immigrant groups using native Swedish-born individuals as referents. This is a retrospective study. The study population included all individuals aged 18 years and older registered in Sweden. PPS was defined as having at least one registered diagnosis in the Swedish National Patient Register. The incidence of post-polio in different immigrant groups, using Swedish-born individuals as referents, was assessed by Cox regression, with hazard ratios (HRs) and 99% confidence intervals (CI). The models were stratified by sex and adjusted for age, geographical residence in Sweden, educational level, marital status, co-morbidities, and neighbourhood socioeconomic status. In total 5300 post-polio cases were registered, 2413 males and 2887 females. Fully adjusted HRs (99% CI) in immigrants versus Swedish-born were 1.77 in men (1.52–2.07) and 1.39 (1.19–1.62) in women. Statistically significant excess risks of post-polio were found in the following subgroups: men and women from Africa, HRs (with 99% CI) 7.40 (5.17–10.59) and 8.39 (5.44–12.95), respectively, and Asia, HRs 6.32 (5.11–7.81) and 4.36 (3.38–5.62) respectively, and in men from Latin America, HR 3.66 (2.17–6.18). It is of importance to be aware of risks of PPS in immigrants settled in Western countries, and that it is more common in immigrants from regions of the world where polio is still prevalent. Patients with PPS need treatment and proper follow-up until polio has been eradicated through global vaccination programs.

## Introduction

Poliomyelitis is an acute viral infection that historically mostly affected children. With improved hygiene, spreading of poliomyelitis will be reduced during childhood, and then also affects individuals later on in life, with an elevated risk of flaccid paresis. The strategy to eradicate polio is expressed by the World Health Organization (WHO) to be “based on preventing infection by immunizing every child until transmission stops and the world is polio-free”^1^.

Owing to vaccination campaigns and better sanitation, the disease has decreased substantially world-wide^2^, but still remains endemic in some countries^1^, i.e. Afghanistan, and Pakistan. Besides, some countries are listed as “outbreak countries”, i.e. that they have stopped indigenous wild poliovirus, but are still experiencing re-infections, e.g. by imported poliovirus, and these include many countries in especially Africa, some countries in the Eastern Mediterranean Region, and also a few European countries^1^. Furthermore, some countries are listed as “key at-risk” countries, mostly many countries in Africa but also China, with low levels of immunity and surveillance and subsequently at risk of polio returning. The last great outbreak of poliomyelitis occurred in Sweden as in other Western countries in the 1950-ies, even if sporadic cases have occurred since then.

Long-term consequences or late effects of poliomyelitis are divided into sequelae of poliomyelitis and post-polio syndrome (PPS). The former is a condition which is non- progressive, and the latter is a condition with progression of increased muscle weakness and muscle atrophy as well as fatigue and pain^3–5^. Europe and other Western countries have given PPS an increased attention during the second half of the twentieth century. The primary criteria necessary for the diagnosis of PPS according to the March of Dimes criteria are: “a history of paralytic poliomyelitis, partial or complete recovery of neurological function followed by a period of stability (usually several decades), persistent new muscle weakness or abnormal muscle fatigability, and the exclusion of other causes of new symptoms”^6^. The prevalence of PPS has been reported to be between 15 and 80% of patients with previous polio, with variation depending on the criteria being used and populations being studied^7^. In Sweden, patients with post-polio have been surveyed, showing an ongoing deterioration^8^, most likely through ongoing neurodegeneration^3^. However, the exact etiology of PPS is not fully established, and there is ongoing controversy and the underlying processes have not been fully elucidated^9^. One study has found a correlation between spinal cord gray matter atrophy and muscle strength, and that this atrophy is associated with functional decline of PPS patients^10^. A study of polio survivors below 60 years of age with foreign background, has been undertaken in Sweden, showing that 50% originated from Asia, 30% from Africa, and only 10% from Europe^11^. Yet, the risk of post-polio in immigrants relative to the risk in the Swedish population or any other Western European country is, as far as we know, largely unknown. We hypothesize that there is an excess risk of post-polio corresponding to those countries and regions of the world where polio is still prevalent today.

The aim of this study was to examine the relative risk of PPS in foreign-born men and women compared to Swedish-born men and women.

## Methods

### Design

We used a retrospective register data design. We used the following national Swedish registers in the present study: the Swedish Total Population Register (TPR), and the Swedish National Patient Register (NPR). These registers are maintained by Statistics Sweden and the National Board of Health and Welfare, and include data on births, deaths, marital status, family relationships, and migration, both within and to/from Sweden, and both these authorities gave administrative decisions to share data for research purposes. The completeness of the Swedish TPR is high, with inclusion of 100% of births and deaths, 95% of immigrations and 91% of emigrations^12^. Regarding the Swedish NPR, including diagnoses reported from hospitals, inpatient hospital care is complete from 1987, with a proportion of missing data of 0.9%, and with more than 99% of all somatic and psychiatric hospital discharges being registered^13^. For out-patient hospital care, diagnoses were included nationwide from 2001 and onwards from specialist care, however not from primary health care, and the rate of missing data is estimated at 3%^14^. The follow-up period ran from January 1, 1998 until hospitalization/out-patient treatment of PPS, death, emigration, or the end of the study period on December 31, 2018, whichever came first.

### Population

The total population of individuals 18 years of age and older in Sweden were included. Immigrants were defined as foreign-born individuals residing in Sweden. Individuals born in Sweden were used as referents and included second-generation immigrants, which had been covered by the national vaccination program.

### Outcome variable

We included the following two diagnoses in the PPS (with ICD-10 code): sequelae of poliomyelitis (B91), and post-polio syndrome (G14).

### Co-morbidities

As co-morbidities we used the following diagnoses (with ICD-10 code): diabetes (E10–E14), hypertension (I10–I19), coronary heart disease (CHD, I20–25), stroke (I60–I69), depressive disorders F32–F33, osteoporotic fractures including humerus fractures (S42.2–S42.4), lower forearm fractures (S52.5 and S52.6), hip fractures (S72.0–S72.2) and vertebral fractures (S12, S22.0, S22.1, S32.0, and T08), and cancer (C00–C97).

### Demographic and socioeconomic variables

The study population was stratified by sex.

Age was used as a continuous variable in the analysis.

Educational attainment (with educational levels for parents for individuals up to 24 years of age) was categorized as ≤ 9 years (partial or complete compulsory schooling), 10–12 years (partial or complete secondary schooling) and > 12 years (attendance at college and/or university).

Marital status was set as married or not (with first noted marital status for parents for individuals up to 24 years of age).

Geographic region of residence was included to adjust for possible regional differences in hospital admissions/specialist care visits and was categorized as (1) large cities, (2) southern Sweden and (3) northern Sweden. Large cities were defined as municipalities with a population of > 2,00,000 and comprised the three largest cities in Sweden: Stockholm, Gothenburg, and Malmö.

### Neighborhood socioeconomic status

Neighborhood socioeconomic status (NSES) was derived from Small Area Market Statistics (SAMS), which were originally created for commercial purposes and pertain to small geographic areas with boundaries defined by homogenous types of buildings. The average population in each SAMS neighborhood is approximately 2000 people for Stockholm and 1000 people for the rest of Sweden. A summary index was calculated to characterize neighborhood-level SES based on four variables: low educational status (< 10 years of formal education); low income (income from all sources, including that from interest and dividends, defined as less than 50% of individual median income); unemployment (not employed, excluding full-time students, those completing compulsory military service, and early retirees); and social welfare payment (receiving social welfare support)^15^. This index was categorized into four groups: more than one standard deviation (SD) below the mean (high SES), more than one SD above the mean (low SES), and within one SD of the mean (moderate SES). The group with high SES was used as reference group. Unknown neighborhood SES comprised its own group.

### Statistical analysis

Baseline data are presented with categorical variables as counts and percentages. We used Cox regression analysis to estimate the relative risk (hazard ratios (HR) with 99% confidence intervals (CI)) of incident PPS in different groups of immigrants compared to Swedish-born individuals during the follow-up time. All analyses were stratified by sex. Three models were used: Model 1 with adjustment for age and region of residence in Sweden; Model 2 with adjustment for age, region of residence, educational level, marital status, and neighborhood SES; and Model 3 as Model 2 but with the inclusion of relevant co-morbidities (diabetes, hypertension, CHD, stroke, depression, osteoporotic fractures, and cancer). We also categorized the PPS into sequelae of poliomyelitis and the postpolio myelitic syndrome, as well as by low and high age into the age-groups 18–60 years and > 60 years of age.

### Ethical approval

All procedures performed in studies involving human participants were in accordance with the ethical standards of the institutional and/or national research committee and with the 1964 Helsinki declaration and its later amendments or comparable ethical standards. Informed consent was not applicable, as the study was based on secondary pseudonymized data from registers. Research data are not shared. The study was approved by the Regional Ethical Review Board in Lund on Feb. 6th, 2013 (ref nr 2012/795 and later amendments).

## Results

The study population (Table 1, Supplementary Tables S1, S2, S3) consisted of 6,180,964 individuals (with 1,050,958 foreign-born, 17.0%), 2,975,068 men (524,594 foreign-born, 17.6%) and 3,205,896 women (526,364 foreign-born, 16.4%). In total, 5300 post-polio cases were registered (993 among foreign-born), 2413 males (538 foreign-born) and 2887 females (455 foreign-born). Among Swedish-born men and women, around 90% were aged 50 years or older, while among foreign-born, around 50% were younger than 40 years of age, i.e. 55% among men and 45% among women (Supplementary Tables 2, 3).

**Table 1:** The study population and the number of cases of post-polio.

	Population		Post polio cases	
	No	%	No	%
Total population	6,180,964		5300	
Gender				
Men	2,975,068	48.1	2413	45.5
Women	3,205,896	51.9	2887	54.5
Immigrant status				
Swedish	5,130,006	83.0	4307	81.3
Foreign-born	1,050,958	17.0	993	18.7
Age (years)				
18–39	2,389,876	38.7	561	10.6
40–49	1,042,598	16.9	590	11.1
50–59	1,067,634	17.3	1465	27.6
60 +	1,680,856	27.2	2684	50.6
Educational level, years				
≤ 9	2,342,469	37.9	1928	36.4
10–12	2,434,806	39.4	2041	38.5
> 12	1,403,689	22.7	1331	25.1
Region of residence				
Large cities	2,731,235	44.2	3019	57.0
Southern Sweden	1,922,900	31.1	1452	27.4
Northern Sweden	1,526,829	24.7	829	15.6
Marital status				
Married	3,491,736	56.5	3591	67.8
Not married	2,689,228	43.5	1709	32.2
Neighborhood deprivation				
Low	1,340,810	21.7	1469	27.7
Middle	3,340,254	54.0	2766	52.2
High	907,919	14.7	795	15.0
Unknown	591,981	9.6	270	5.1
Hospital diagnoses				
Diabetes	462,887	7.5	642	12.1
Coronary heart disease	1,223,125	19.8	1785	33.7
Hypertension	1,126,486	18.2	1776	33.5
Stroke	494,487	8.0	858	16.2
Depression	289,836	4.7	315	5.9
Osteoporotic fractures	334,944	5.4	491	9.3
Cancer	1,120,363	18.1	1665	31.4
All	6,180,964	100.0	5300	100.0

As regards background factors (Supplementary Tables S4, S5), the risk was higher in Swedish-born men and women for the two higher educational levels, and also for those being married. Regarding region of residence, the risk was lower in the southern and northern region of Sweden for Swedish-born men, and for both Swedish-born and foreign-born women, when compared to women and men residing in the large Swedish cities. For NSES the HRs were lower only among Swedish-born women living in middle NSES or unknown NSES levels (Supplementary Table S5). Regarding co-morbidities, for Swedish-born men the HRs were higher for hypertension, stroke, depression, and cancer; for foreign-born men for stroke; for Swedish-born women for hypertension and cancer. For foreign-born women no statistically significant values were noted.

The incidence rates by 100,000 person-years are shown in Table 2. Among all men, it was 1.96 and among all women 2.08 per 100,000 person-years in 1998–2018. The corresponding incidence rates among Swedish-born were 1.48 and 1.72 in men and women, respectively, and among foreign-born men and women 4.32 and 3.61, respectively. The risk of being diagnosed with incident PPS by sex in the different immigrant groups compared to Swedish-born is shown in Table 3. Overall, the risk was higher in immigrant men and women. For the different immigrant groups, the risk was higher in men and women from Africa and Asia, and also in men from Latin America, in all three models. The risk was lower in men from the Nordic countries. For sequelae of poliomyelitis (Table 4), the risk pattern was similar to the overall PPS, but for the PPS the risk was higher only among men and women from Asia, and men from Africa. Categorization into lower and higher age (Table 5) showed a higher risk among men and women ≤ 60 years of age overall among immigrants, and in men and women from Africa, Latin America, and Asia; a lower risk among men from the Nordic countries was found in both lower and higher ages.

**Table 2:** Incidence rate (per 100, 000 person years) of post-polio in the first-generation individuals, 1998–2018.

	All				Swedish				Foreign born			
	No	Rate*	95% CI		No	Rate*	95% CI		No	Rate*	95% CI	
Men	2413	1.96	1.88	2.04	1875	1.48	1.41	1.55	538	4.32	3.95	4.69
Women	2887	2.05	1.98	2.12	2432	1.72	1.65	1.79	455	3.61	3.28	3.94

*Adjusted for World standardized population.

**Table 3:** The relative risk of post-polio in immigrants vs Swedish-born individuals expressed as hazard ratios (HRs) with 99% confidence intervals (99% CI).

	Obs	Model 1			Model 2			Model 3		
		HR	99% CI		HR	99% CI		HR	99% CI	
Men										
Sweden	1875	1			1			1		
All foreign-born	538	**1.76**	**1.53**	**2.03**	**1.79**	**1.53**	**2.09**	**1.77**	**1.52**	**2.07**
Nordic countries	61	**0.51**	**0.35**	**0.74**	**0.57**	**0.39**	**0.84**	**0.58**	**0.40**	**0.85**
Southern Europe	10	0.49	0.20	1.21	0.53	0.22	1.33	0.56	0.23	1.39
Western Europe	14	0.55	0.26	1.18	0.53	0.24	1.13	0.53	0.25	1.14
Eastern Europe	27	0.86	0.50	1.50	0.85	0.49	1.49	0.85	0.49	1.49
Baltic countries	8	1.28	0.46	3.50	1.14	0.41	3.12	1.12	0.41	3.06
Central Europe	16	0.95	0.46	1.95	0.86	0.42	1.77	0.85	0.41	1.74
Africa	73	**6.85**	**4.84**	**9.68**	**7.29**	**5.10**	**10.42**	**7.40**	**5.17**	**10.59**
Northern America	9	1.34	0.52	3.47	1.44	0.55	3.73	1.47	0.57	3.83
Latin America	31	**3.45**	**2.05**	**5.79**	**3.53**	**2.09**	**5.96**	**3.66**	**2.17**	**6.18**
Asia	286	**6.09**	**5.03**	**7.37**	**6.33**	**5.13**	**7.81**	**6.32**	**5.11**	**7.81**
Russia	2	0.68	0.09	5.10	0.69	0.09	5.15	0.69	0.09	5.18
Women										
Sweden	2432	1			1			1		
All foreign-born:	455	**1.30**	**1.12**	**1.50**	**1.40**	**1.20**	**1.63**	**1.39**	**1.19**	**1.62**
Nordica countries	135	0.81	0.63	1.04	0.92	0.71	1.19	0.91	0.70	1.17
Southern Europe	10	0.72	0.29	1.77	0.92	0.37	2.28	0.95	0.38	2.36
Western Europe	27	0.95	0.55	1.65	0.94	0.54	1.64	0.92	0.53	1.60
Eastern Europe	18	0.61	0.31	1.20	0.65	0.33	1.28	0.66	0.33	1.29
Baltic countries	5	0.61	0.17	2.19	0.56	0.16	2.01	0.55	0.15	1.98
Central Europe	22	1.05	0.57	1.93	1.00	0.54	1.84	0.99	0.54	1.82
Africa	48	**6.57**	**4.30**	**10.04**	**8.18**	**5.31**	**12.61**	**8.39**	**5.44**	**12.95**
Northern America	7	1.02	0.35	3.01	1.21	0.41	3.55	1.23	0.42	3.61
Latin America	15	1.53	0.73	3.20	1.70	0.81	3.57	1.74	0.83	3.66
Asia	165	**3.69**	**2.90**	**4.69**	**4.30**	**3.34**	**5.54**	**4.36**	**3.38**	**5.62**
Russia	2	0.41	0.05	3.08	0.45	0.06	3.35	0.45	0.06	3.34

Bold values denotes statistically significant values.

Model 1: adjusted for age and region of residence in Sweden; model 2: adjusted for age, region of residence in Sweden, educational level, marital status, and neighobrhood deprivations; model 3: model 2 + comorbidities.

**Table 4:** The relative risk of post-polio in immigrants vs Swedish-born individuals categorized into sequelae or post-polio syndrome expressed as hazard ratios (HRs) with 99% confidence intervals (99% CI).

	Sequelae of poliomyelitis			Postpolio syndrome		
	HR*	99% CI		HR*	99% CI	
Males						
Sweden	1			1		
All foreign-born	1.77	1.50	2.09	1.77	1.11	2.82
Nordic countries	0.62	0.42	0.93	0.33	0.10	1.13
Southern Europe	0.41	0.13	1.32	1.17	0.27	5.20
Western Europe	0.57	0.25	1.25	0.28	0.02	5.01
Eastern Europe	0.84	0.46	1.53	0.91	0.21	3.91
Baltic countries	1.21	0.44	3.32	–		
Central Europe	0.84	0.39	1.80	0.99	0.13	7.57
Africa	7.55	5.16	11.03	5.30	1.78	15.71
Northern America	1.71	0.66	4.45	–		
Latin America	4.00	2.33	6.88	1.50	0.19	11.64
Asia	6.11	4.84	7.69	7.24	4.12	12.71
Russia	0.38	0.02	6.65	2.93	0.17	51.72
Females						
Sweden	1			1		
All foreign-born	1.38	1.17	1.62	1.46	0.84	2.51
Nordic countries	0.93	0.71	1.21	0.66	0.24	1.78
Southern Europe	0.95	0.37	2.49	0.83	0.05	15.18
Western Europe	0.86	0.47	1.56	1.54	0.36	6.60
Eastern Europe	0.59	0.28	1.25	1.43	0.27	7.66
Baltic countries	0.59	0.17	2.13	–		
Central Europe	0.92	0.48	1.78	1.78	0.34	9.43
Africa	8.49	5.44	13.24	5.74	0.73	45.20
Northern America	1.14	0.36	3.67	1.86	0.10	33.19
Latin America	1.89	0.90	3.98	–		
Asia	4.27	3.27	5.58	5.95	2.55	13.84
Russia	0.48	0.06	3.58	–		

Bold values denotes statistically significant values.

*Fully adjusted.

**Table 5:** The relative risk of post-polio in immigrants vs Swedish-born individuals categorized by age expressed as hazard ratios (HRs) with 99% confidence intervals (99% CI).

	≤ 60 years			> 60 years		
	HR*	99% CI		HR*	99% CI	
Males						
Sweden	1			1		
All foreign-born	**2.64**	**2.18**	**3.19**	**0.47**	**0.31**	**0.70**
Nordic countries	**0.58**	**0.34**	**0.96**	**0.54**	**0.31**	**0.95**
Southern Europe	0.75	0.27	2.08	0.30	0.04	2.23
Western Europe	0.59	0.20	1.73	0.43	0.15	1.28
Eastern Europe	1.24	0.71	2.19	–		
Baltic countries	1.88	0.36	9.75	0.85	0.24	3.04
Central Europe	1.49	0.69	3.23	0.20	0.03	1.47
Africa	**8.75**	**6.01**	**12.74**	0.76	0.04	13.11
Northern America	1.33	0.32	5.59	1.67	0.46	6.01
Latin America	**4.64**	**2.73**	**7.90**	–		
Asia	**8.16**	**6.43**	**10.36**	0.39	0.07	2.05
Russia	0.79	0.05	13.64	0.64	0.04	11.12
Females						
Sweden				1		
All foreign-born	**1.87**	**1.55**	**2.27**	**0.67**	**0.50**	**0.91**
Nordic countries	0.84	0.59	1.20	0.73	0.50	1.05
Southern Europe	1.35	0.51	3.54	0.21	0.01	3.64
Western Europe	0.72	0.26	1.98	0.80	0.41	1.54
Eastern Europe	0.80	0.38	1.68	0.42	0.08	2.19
Baltic countries	–			0.73	0.20	2.62
Central Europe	1.48	0.77	2.81	0.24	0.03	1.77
Africa	**14.28**	**9.12**	**22.36**	–		
Northern America	–			2.14	0.73	6.32
Latin America	**02.30**	**1.09**	**4.85**	–		
Asia	**7.08**	**5.38**	**9.32**	0.20	0.01	3.42
Russia	1.01	0.13	7.63	–		

*Fully adjusted.

Bold values denotes statistically significant values.

## Discussion

The main results of the study were that the risk of being diagnosed with incident PPS was higher in both men and women from Africa and Asia, and in men from Latin America. This pattern was also seen among those with the diagnosis sequelae of poliomyelitis, and among those ≤ 60 years of age. Among foreign-born women and men, 45–55% were younger than 40 years, while among Swedish-born individuals 90% were aged 50 years or older.

A higher relative risk in immigrants from non-Western regions are in line with our hypothesis and with earlier results^11^, as polio has been present to a higher extent in those regions of the world, while polio is rather rare in Western regions, i.e., Europe and North America ever since the vaccination programs started in the 50’ies and 60’ies. The risk of post-polio was lower among foreign-born from the Nordic countries only, perhaps linked to the “healthy migrant effect”, i.e. individuals who emigrate tend to be healthier than their compatriots in the country of origin^16^. The healthy migrant effect could also explain the lack of significant findings in immigrant groups from many other countries, as individuals with polio and post-polio may be less likely to leave their home countries.

Identifying patients with PPS is important, to be able to give treatment, even if no specific treatment exists^3^. However, physical activity, physiotherapy, and muscle training are the basis of the management of PPS patients, but assistance from other clinical specialists could also be used^9^. Surgery, orthosis, and assistive devices could be useful based on the specific individual needs. In the study by Werhagen and Borg^11^, it was reported that nonnative patients, i.e. patients contracting polio outside Sweden showed similarities in age at the acute polio infection, type of paresis, prevalence, and intensity of pain when compared with native Swedish polio patients. They were, however, less often working and married/cohabiting than native Swedish polio survivors. Werhagen and Borg called for tailored rehabilitation suitable for this group of polio patients^11^, which is further underlined by the results of the present study.

Regarding background factors, living in the three large Swedish cities, Stockholm Gothenburg, and Malmö, was associated with a higher risk of incident PPS, which could be explained by the fact that many immigrants live in the larger cities. It is also a higher possibility of a contact with a neurologist in individuals residing in larger cities. and probably also a higher awareness of the post-polio-syndrome. The risk of post-polio was also higher for Swedish-born individuals with higher education, which could possibly also be associated with a higher educational level in the large cities, as well as higher awareness and access to neurologists.

Among the co-morbidities, we found higher risks of post-polio for Swedish-born men with hypertension, stroke, depression, and cancer and for foreign-born men for stroke. The higher risk of stroke in patients with polio and with subsequent hypertension has been reported previously^17^, but we found that this increased risk was only present in men. Regarding depression^18^, we only found an excess risk among Swedish-born men. An increased risk of cancer is described among aging adults with chronic long-term physical disability^19^, not specifically for PPS patients. In opposite to other studies we found no association between PPS and CHD^18^, or osteoporotic fractures^20^.

It is important for public health planners to be aware of that polio still exists in the world, and to continue to vaccinate Swedish children, and that post-polio cases will continue to emerge in Sweden. The anti-vaccination groups in Sweden have increased after the narcolepsy cases after the Pandemrix vaccination against the H1N1 influenza in 2009–2010^21^. However, the coverage of polio-vaccination in Sweden 2021, i.e. during the Covid-19 pandemic, was 97% and no decrease in coverage was reported during recent years according to statistics from the Public Health Agency of Sweden^22^.

As polio spread through contaminated water, improved sanitation is of importance in regions of the world where polio is still present. Potential threats to the global vaccination program and antivaccination campaigns as well as sanitation for all people will, as shown in this present study, not only affect the health of the population in those regions of the world with prevalent polio, but also lead to suffering and healthcare costs in Western countries.

This study has limitations. We used register data, with no possibility to check clinical data and the validity and criteria of the diagnoses, and there is a risk that diagnostic criteria are not applied uniformly by various neurologists at different centers. We could not identify the date of the primary polio diagnosis. To catch all cases with PPSs, patients must have searched specialist care, as patients visiting primary care are not included in the Swedish NPR. However, at a suspected diagnosis of PPS, patients should normally be referred to a neurologist or a special rehabilitation unit. Of course, patients not seeking care, or doctors not recognizing post-polio symptoms in the patients, would be missed, and it is possible that this occurs to a higher extent in immigrants due to language and cultural barriers.

The last registered case of polio in Sweden was reported in 1977. Different explanations for the number of patients born in Sweden, aged 18–39 years, and reported to have PPS may be explained by the immigration to Sweden in the 1950:s and 1960:s, i.e. patients born in Sweden but contracting polio when visiting their parents’ home country before being fully vaccinated. As there is no opportunity to further evaluate the clinical diagnosis from nationwide registers one cannot rule out that there may be errors in the reporting of the disorder to the Swedish National Patient Register.

There are several strengths of the study. The national Swedish registers are known to be of high quality, such as the NPR and the TPR^12,13^. The Swedish personal identity number also enables secure linkage between Swedish registers^23^, providing high integrity for the individuals by the use of pseudonymized data.

In conclusion, we found a higher risk of PPSs in individuals from non-Western regions, especially from Africa and Asia. It is of importance to be aware of PPS in the whole healthcare system in Western countries, and that it is more common in immigrants from regions of the world where polio is still prevalent. PPS patients need treatment and follow-up for their lifetime. PPS will only disappear decades after the worldwide eradication of polio.

## Data availability 

The datasets generated and/or analyzed during the current study are not publicly available due to restraints in the ethical approvals but we are willing to collaborate upon request to the corresponding author, axel.carlsson@ki.se.

## Supplementary Information

Supplementary Information.
