# Supplementary material for: The risk of post-polio syndrome among immigrant groups in Sweden
Source: Sci Rep. 2023 Apr 13;13:6044. doi: 10.1038/s41598-023-33240-w (PMC10098995; doi:10.1038/s41598-023-33240-w)
Supplement: Supplementary file 1 — Supplementary Information. [file 41598_2023_33240_MOESM1_ESM.docx]

| **Supplementary Table S1. Study population and the number of post-polio syndrome cases categorized by sex** | | | | | | | | | | | | | | | | |
| --- | --- | --- | --- | --- | --- | --- | --- | --- | --- | --- | --- | --- | --- | --- | --- | --- |
|  | Men | | | | | |  | | Women | | | | | | | |
|  | Population | |  | Post-polio cases | |  | | Population | | |  | | Post-polio cases | | |  |
|  | No. | % |  | No | % | |  | | No. | % | |  | | No | % | |
| Total population | 2975068 |  |  | 2413 |  | |  | | 3205896 |  | |  | | 2887 |  | |
| Immigrant status |  |  |  |  |  | |  | |  |  | |  | |  |  | |
| Swedish | 2450474 | 82.4 |  | 1875 | 77.7 | |  | | 2679532 | 83.6 | |  | | 2432 | 84.2 | |
| Foreign born | 524594 | 17.6 |  | 538 | 22.3 | |  | | 526364 | 16.4 | |  | | 455 | 15.8 | |
| Age (years) |  | 0.0 |  |  | 0.0 | |  | |  | 0.0 | |  | |  | 0.0 | |
| 18-39 | 1175475 | 39.5 |  | 329 | 13.6 | |  | | 1214401 | 37.9 | |  | | 232 | 8.0 | |
| 40-49 | 514992 | 17.3 |  | 283 | 11.7 | |  | | 527606 | 16.5 | |  | | 307 | 10.6 | |
| 50-59 | 532531 | 17.9 |  | 633 | 26.2 | |  | | 535103 | 16.7 | |  | | 832 | 28.8 | |
| 60+ | 752070 | 25.3 |  | 1168 | 48.4 | |  | | 928786 | 29.0 | |  | | 1516 | 52.5 | |
| Educational level |  |  |  |  |  | |  | |  |  | |  | |  |  | |
| ≤ 9 | 1126191 | 37.9 |  | 817 | 33.9 | |  | | 1216278 | 37.9 | |  | | 1111 | 38.5 | |
| 10-12 | 1189595 | 40.0 |  | 948 | 39.3 | |  | | 1245211 | 38.8 | |  | | 1093 | 37.9 | |
| > 12 | 659282 | 22.2 |  | 648 | 26.9 | |  | | 744407 | 23.2 | |  | | 683 | 23.7 | |
| Region of residence |  |  |  |  |  | |  | |  |  | |  | |  |  | |
| Large cities | 1299198 | 43.7 |  | 1348 | 55.9 | |  | | 1432037 | 44.7 | |  | | 1671 | 57.9 | |
| Southern Sweden | 916298 | 30.8 |  | 643 | 26.6 | |  | | 1006602 | 31.4 | |  | | 809 | 28.0 | |
| Northern Sweden | 759572 | 25.5 |  | 422 | 17.5 | |  | | 767257 | 23.9 | |  | | 407 | 14.1 | |
| Marital status |  |  |  |  |  | |  | |  |  | |  | |  |  | |
| Married | 1765268 | 59.3 |  | 1825 | 75.6 | |  | | 1726468 | 53.9 | |  | | 1766 | 61.2 | |
| Not married | 1209800 | 40.7 |  | 588 | 24.4 | |  | | 1479428 | 46.1 | |  | | 1121 | 38.8 | |
| Neighborhood deprivation |  |  |  |  |  | |  | |  |  | |  | |  |  | |
| Low | 642443 | 21.6 |  | 652 | 27.0 | |  | | 698367 | 21.8 | |  | | 817 | 28.3 | |
| Middle | 1585960 | 53.3 |  | 1210 | 50.1 | |  | | 1754294 | 54.7 | |  | | 1556 | 53.9 | |
| High | 430820 | 14.5 |  | 371 | 15.4 | |  | | 477099 | 14.9 | |  | | 424 | 14.7 | |
| Unknown | 315845 | 10.6 |  | 180 | 7.5 | |  | | 276136 | 8.6 | |  | | 90 | 3.1 | |
| Diagnosis of diabetes |  |  |  |  |  | |  | |  |  | |  | |  |  | |
| Non | 2721088 | 91.5 |  | 2078 | 86.1 | |  | | 2996989 | 93.5 | |  | | 2580 | 89.4 | |
| Yes | 253980 | 8.5 |  | 335 | 13.9 | |  | | 208907 | 6.5 | |  | | 307 | 10.6 | |
| Diagnosis of coronary heart disease |  |  |  |  |  | |  | |  |  | |  | |  |  | |
| Non | 2302898 | 77.4 |  | 1488 | 61.7 | |  | | 2654941 | 82.8 | |  | | 2027 | 70.2 | |
| Yes | 672170 | 22.6 |  | 925 | 38.3 | |  | | 550955 | 17.2 | |  | | 860 | 29.8 | |
| Diagnosis of hypertension |  |  |  |  |  | |  | |  |  | |  | |  |  | |
| Non | 2435096 | 81.9 |  | 1624 | 67.3 | |  | | 2619382 | 81.7 | |  | | 1900 | 65.8 | |
| Yes | 539972 | 18.1 |  | 789 | 32.7 | |  | | 586514 | 18.3 | |  | | 987 | 34.2 | |
| Diagnosis of stroke |  |  |  |  |  | |  | |  |  | |  | |  |  | |
| Non | 2724549 | 91.6 |  | 1978 | 82.0 | |  | | 2961928 | 92.4 | |  | | 2464 | 85.3 | |
| Yes | 250519 | 8.4 |  | 435 | 18.0 | |  | | 243968 | 7.6 | |  | | 423 | 14.7 | |
| Diagnosis of depression |  |  |  |  |  | |  | |  |  | |  | |  |  | |
| Non | 2864911 | 96.3 |  | 2278 | 94.4 | |  | | 3026217 | 94.4 | |  | | 2707 | 93.8 | |
| Yes | 110157 | 3.7 |  | 135 | 5.6 | |  | | 179679 | 5.6 | |  | | 180 | 6.2 | |
| Diagnosis of fracture |  |  |  |  |  | |  | |  |  | |  | |  |  | |
| Non | 2879679 | 96.8 |  | 2297 | 95.2 | |  | | 2966341 | 92.5 | |  | | 2512 | 87.0 | |
| Yes | 95389 | 3.2 |  | 116 | 4.8 | |  | | 239555 | 7.5 | |  | | 375 | 13.0 | |
| Diagnosis of cancer |  |  |  |  |  | |  | |  |  | |  | |  |  | |
| Non | 2417889 | 81.3 |  | 1602 | 66.4 | |  | | 2642712 | 82.4 | |  | | 2033 | 70.4 | |
| Yes | 557179 | 18.7 |  | 811 | 33.6 | |  | | 563184 | 17.6 | |  | | 854 | 29.6 | |
| All | 2975068 | 100.0 |  | 2413 | 100.0 | |  | | 3205896 | 100.0 | |  | | 2887 | 100.0 | |

| **Supplementary Table S2. Study population and the number of post-polio cases in men** | | | | | | | | | | | | |
| --- | --- | --- | --- | --- | --- | --- | --- | --- | --- | --- | --- | --- |
|  | Swedish born | | | | |  | Foreign born | | | | | |
|  | Population | |  | Post-polio cases | |  | Population | | |  | Post-polio cases | |
|  | No. | % |  | No | % |  | No. | % |  | | No | % |
| Total population | 2450474 |  |  | 1875 |  |  | 524594 |  |  | | 538 |  |
| Age (years) |  |  |  |  |  |  |  |  |  | |  |  |
| 18-39 | 908502 | 37.1 |  | 29 | 1.5 |  | 266973 | 50.9 |  | | 300 | 55.8 |
| 40-49 | 419062 | 17.1 |  | 162 | 8.6 |  | 95930 | 18.3 |  | | 121 | 22.5 |
| 50-59 | 449403 | 18.3 |  | 573 | 30.6 |  | 83128 | 15.8 |  | | 60 | 11.2 |
| 60+ | 673507 | 27.5 |  | 1111 | 59.3 |  | 78563 | 15.0 |  | | 57 | 10.6 |
| Educational level |  |  |  |  |  |  |  |  |  | |  |  |
| ≤ 9 | 810292 | 33.1 |  | 559 | 29.8 |  | 315899 | 60.2 |  | | 258 | 48.0 |
| 10-12 | 1064625 | 43.4 |  | 796 | 42.5 |  | 124970 | 23.8 |  | | 152 | 28.3 |
| > 12 | 575557 | 23.5 |  | 520 | 27.7 |  | 83725 | 16.0 |  | | 128 | 23.8 |
| Region of residence |  |  |  |  |  |  |  |  |  | |  |  |
| Large cities | 1107521 | 45.2 |  | 1081 | 57.7 |  | 191677 | 36.5 |  | | 267 | 49.6 |
| Southern Sweden | 830226 | 33.9 |  | 554 | 29.5 |  | 86072 | 16.4 |  | | 89 | 16.5 |
| Northern Sweden | 512727 | 20.9 |  | 240 | 12.8 |  | 246845 | 47.1 |  | | 182 | 33.8 |
| Marital status |  |  |  |  |  |  |  |  |  | |  |  |
| Married | 1359807 | 55.5 |  | 1431 | 76.3 |  | 405461 | 77.3 |  | | 394 | 73.2 |
| Not married | 1090667 | 44.5 |  | 444 | 23.7 |  | 119133 | 22.7 |  | | 144 | 26.8 |
| Neighborhood deprivation |  |  |  |  |  |  |  |  |  | |  |  |
| Low | 591604 | 24.1 |  | 586 | 31.3 |  | 50839 | 9.7 |  | | 66 | 12.3 |
| Middle | 1448921 | 59.1 |  | 1054 | 56.2 |  | 137039 | 26.1 |  | | 156 | 29.0 |
| High | 317040 | 12.9 |  | 212 | 11.3 |  | 113780 | 21.7 |  | | 159 | 29.6 |
| Unknown | 92909 | 3.8 |  | 23 | 1.2 |  | 222936 | 42.5 |  | | 157 | 29.2 |
| Diagnosis of diabetes |  |  |  |  |  |  |  |  |  | |  |  |
| Non | 2237526 | 91.3 |  | 1612 | 86.0 |  | 483562 | 92.2 |  | | 466 | 86.6 |
| Yes | 212948 | 8.7 |  | 263 | 14.0 |  | 41032 | 7.8 |  | | 72 | 13.4 |
| Diagnosis of coronary heart disease |  |  |  |  |  |  |  |  |  | |  |  |
| Non | 1853904 | 75.7 |  | 1053 | 56.2 |  | 448994 | 85.6 |  | | 435 | 80.9 |
| Yes | 596570 | 24.3 |  | 822 | 43.8 |  | 75600 | 14.4 |  | | 103 | 19.1 |
| Diagnosis of hypertension |  |  |  |  |  |  |  |  |  | |  |  |
| Non | 1976369 | 80.7 |  | 1195 | 63.7 |  | 458727 | 87.4 |  | | 429 | 79.7 |
| Yes | 474105 | 19.3 |  | 680 | 36.3 |  | 65867 | 12.6 |  | | 109 | 20.3 |
| Diagnosis of stroke |  |  |  |  |  |  |  |  |  | |  |  |
| Non | 2225719 | 90.8 |  | 1487 | 79.3 |  | 498830 | 95.1 |  | | 491 | 91.3 |
| Yes | 224755 | 9.2 |  | 388 | 20.7 |  | 25764 | 4.9 |  | | 47 | 8.7 |
| Diagnosis of depression |  |  |  |  |  |  |  |  |  | |  |  |
| Non | 2361892 | 96.4 |  | 1781 | 95.0 |  | 503019 | 95.9 |  | | 497 | 92.4 |
| Yes | 88582 | 3.6 |  | 94 | 5.0 |  | 21575 | 4.1 |  | | 41 | 7.6 |
| Diagnosis of fracture |  |  |  |  |  |  |  |  |  | |  |  |
| Non | 2365654 | 96.5 |  | 1774 | 94.6 |  | 514025 | 98.0 |  | | 523 | 97.2 |
| Yes | 84820 | 3.5 |  | 101 | 5.4 |  | 10569 | 2.0 |  | | 15 | 2.8 |
| Diagnosis of cancer |  |  |  |  |  |  |  |  |  | |  |  |
| Non | 1946605 | 79.4 |  | 1134 | 60.5 |  | 471284 | 89.8 |  | | 468 | 87.0 |
| Yes | 503869 | 20.6 |  | 741 | 39.5 |  | 53310 | 10.2 |  | | 70 | 13.0 |
| All | 2450474 | 100.0 |  | 1875 | 100.0 |  | 524594 | 100.0 |  | | 538 | 100.0 |

| **Supplementary Table S3. Study population and the number of post-polio cases in women** | | | | | | | | | | |  |
| --- | --- | --- | --- | --- | --- | --- | --- | --- | --- | --- | --- |
|  | Swedish born | | | | |  | Foreign born | | | | |
|  | Population | |  | Post-polio cases | |  | Population | |  | Post-polio cases | |
|  | No. | % |  | No | % |  | No. | % |  | No | % |
| Total population | 2679532 |  |  | 2432 |  |  | 526364 |  |  | 455 |  |
| Age (years) |  |  |  |  |  |  |  |  |  |  |  |
| 18-39 | 941902 | 35.2 |  | 24 | 1.0 |  | 272499 | 51.8 |  | 208 | 45.7 |
| 40-49 | 433322 | 16.2 |  | 230 | 9.5 |  | 94284 | 17.9 |  | 77 | 16.9 |
| 50-59 | 463081 | 17.3 |  | 769 | 31.6 |  | 72022 | 13.7 |  | 63 | 13.8 |
| 60+ | 841227 | 31.4 |  | 1409 | 57.9 |  | 87559 | 16.6 |  | 107 | 23.5 |
| Educational level |  |  |  |  |  |  |  |  |  |  |  |
| ≤ 9 | 904313 | 33.7 |  | 920 | 37.8 |  | 311965 | 59.3 |  | 191 | 42.0 |
| 10-12 | 1119925 | 41.8 |  | 937 | 38.5 |  | 125286 | 23.8 |  | 156 | 34.3 |
| > 12 | 655294 | 24.5 |  | 575 | 23.6 |  | 89113 | 16.9 |  | 108 | 23.7 |
| Region of residence |  |  |  |  |  |  |  |  |  |  |  |
| Large cities | 1226537 | 45.8 |  | 1393 | 57.3 |  | 205500 | 39.0 |  | 278 | 61.1 |
| Southern Sweden | 913828 | 34.1 |  | 727 | 29.9 |  | 92774 | 17.6 |  | 82 | 18.0 |
| Northern Sweden | 539167 | 20.1 |  | 312 | 12.8 |  | 228090 | 43.3 |  | 95 | 20.9 |
| Marital status |  |  |  |  |  |  |  |  |  |  |  |
| Married | 1336190 | 49.9 |  | 1470 | 60.4 |  | 390278 | 74.1 |  | 296 | 65.1 |
| Not married | 1343342 | 50.1 |  | 962 | 39.6 |  | 136086 | 25.9 |  | 159 | 34.9 |
| Neighborhood deprivation |  |  |  |  |  |  |  |  |  |  |  |
| Low | 637552 | 23.8 |  | 728 | 29.9 |  | 60815 | 11.6 |  | 89 | 19.6 |
| Middle | 1598150 | 59.6 |  | 1379 | 56.7 |  | 156144 | 29.7 |  | 177 | 38.9 |
| High | 363894 | 13.6 |  | 312 | 12.8 |  | 113205 | 21.5 |  | 112 | 24.6 |
| Unknown | 79936 | 3.0 |  | 13 | 0.5 |  | 196200 | 37.3 |  | 77 | 16.9 |
| Diagnosis of diabetes |  |  |  |  |  |  |  |  |  |  |  |
| Non | 2500740 | 93.3 |  | 2164 | 89.0 |  | 496249 | 94.3 |  | 416 | 91.4 |
| Yes | 178792 | 6.7 |  | 268 | 11.0 |  | 30115 | 5.7 |  | 39 | 8.6 |
| Diagnosis of coronary heart disease |  |  |  |  |  |  |  |  |  |  |  |
| Non | 2185756 | 81.6 |  | 1646 | 67.7 |  | 469185 | 89.1 |  | 381 | 83.7 |
| Yes | 493776 | 18.4 |  | 786 | 32.3 |  | 57179 | 10.9 |  | 74 | 16.3 |
| Diagnosis of hypertension |  |  |  |  |  |  |  |  |  |  |  |
| Non | 2163925 | 80.8 |  | 1544 | 63.5 |  | 455457 | 86.5 |  | 356 | 78.2 |
| Yes | 515607 | 19.2 |  | 888 | 36.5 |  | 70907 | 13.5 |  | 99 | 21.8 |
| Diagnosis of stroke |  |  |  |  |  |  |  |  |  |  |  |
| Non | 2459416 | 91.8 |  | 2046 | 84.1 |  | 502512 | 95.5 |  | 418 | 91.9 |
| Yes | 220116 | 8.2 |  | 386 | 15.9 |  | 23852 | 4.5 |  | 37 | 8.1 |
| Diagnosis of depression |  |  |  |  |  |  |  |  |  |  |  |
| Non | 2532627 | 94.5 |  | 2288 | 94.1 |  | 493590 | 93.8 |  | 419 | 92.1 |
| Yes | 146905 | 5.5 |  | 144 | 5.9 |  | 32774 | 6.2 |  | 36 | 7.9 |
| Diagnosis of fracture |  |  |  |  |  |  |  |  |  |  |  |
| Non | 2463399 | 91.9 |  | 2085 | 85.7 |  | 502942 | 95.6 |  | 427 | 93.8 |
| Yes | 216133 | 8.1 |  | 347 | 14.3 |  | 23422 | 4.4 |  | 28 | 6.2 |
| Diagnosis of cancer |  |  |  |  |  |  |  |  |  |  |  |
| Non | 2171599 | 81.0 |  | 1655 | 68.1 |  | 471113 | 89.5 |  | 378 | 83.1 |
| Yes | 507933 | 19.0 |  | 777 | 31.9 |  | 55251 | 10.5 |  | 77 | 16.9 |
| All | 2679532 | 100.0 |  | 2432 | 100.0 |  | 526364 | 100.0 |  | 455 | 100.0 |

| **Supplementary Table S4. The relative risk of post-polio in immigrant men vs Swedish-born men expressed as hazard ratios (HR) with 99% confidence intervals (99% CI)** | | | | | | | | |
| --- | --- | --- | --- | --- | --- | --- | --- | --- |
|  | Swedish born | | |  | Foreign born | | |  |
|  | HR* | 99% CI | |  | HR* | 99% CI | |  |
| Birth year | **0.94** | **0.94** | **0.95** |  | **1.02** | **1.01** | **1.03** |  |
| Education level (ref. <9 years) |  |  |  |  |  |  |  |  |
| 10-12 | **1.63** | **1.38** | **1.92** |  | 1.09 | 0.76 | 1.57 |  |
| > 12 | **1.68** | **1.40** | **2.02** |  | 1.41 | 0.96 | 2.07 |  |
| Region of residence (ref. Large cities) |  |  |  |  |  |  |  |  |
| Southern Sweden | **0.71** | **0.61** | **0.82** |  | 0.76 | 0.54 | 1.08 |  |
| Northern Sweden | **0.55** | **0.44** | **0.68** |  | 0.80 | 0.44 | 1.44 |  |
| Marital status (ref. Married) | **1.30** | **1.11** | **1.52** |  | 1.16 | 0.85 | 1.59 |  |
| Neighborhood deprivation (ref. Low) |  |  |  |  |  |  |  |  |
| Middle | 0.86 | 0.73 | 1.00 |  | 0.93 | 0.61 | 1.42 |  |
| High | 0.84 | 0.67 | 1.07 |  | 1.03 | 0.67 | 1.57 |  |
| Unknown | 0.59 | 0.30 | 1.16 |  | 0.91 | 0.42 | 1.96 |  |
| Diagnosis of diabetes (ref. Non) | 1.08 | 0.89 | 1.31 |  | 1.44 | 0.97 | 2.12 |  |
| Diagnosis of coronary heart disease (ref. Non) | 1.00 | 0.86 | 1.16 |  | 1.15 | 0.79 | 1.68 |  |
| Diagnosis of hypertension (ref. Non) | **1.25** | **1.08** | **1.45** |  | 1.35 | 0.94 | 1.95 |  |
| Diagnosis of stroke (ref. Non) | **1.23** | **1.04** | **1.46** |  | **1.68** | **1.05** | **2.71** |  |
| Diagnosis of depression (ref. Non) | **1.51** | **1.11** | **2.04** |  | 1.44 | 0.90 | 2.30 |  |
| Diagnosis of fracture (ref. Non) | 1.04 | 0.78 | 1.40 |  | 1.12 | 0.53 | 2.37 |  |
| Diagnosis of cancer (ref. Non) | **1.29** | **1.12** | **1.48** |  | 1.31 | 0.88 | 1.94 |  |
| HR: Hazard ratios; CI: confidence interval. | | | | | | |  |  |
| *: Full adjusted. |  |  |  |  |  |  |  |  |

Bold values are statistically significant.

| **Supplementary Table S5. The relative risk of post-polio in immigrant women vs Swedish-born women expressed as hazard ratios (HR) with 99% confidence intervals (99% CI)** | | | | | | | | |
| --- | --- | --- | --- | --- | --- | --- | --- | --- |
|  | Swedish born | | |  | Foreign born | | |  |
|  | HR* | 99% CI | |  | HR* | 99% CI | |  |
| Birth year | **0.95** | **0.95** | **0.96** |  | 1.00 | 0.99 | 1.01 |  |
| Education level (ref. <9 years) |  |  |  |  |  |  |  |  |
| 10-12 | **1.33** | **1.16** | **1.53** |  | 1.22 | 0.85 | 1.74 |  |
| > 12 | **1.33** | **1.13** | **1.56** |  | 1.22 | 0.82 | 1.81 |  |
| Region of residence (ref. Large cities) |  |  |  |  |  |  |  |  |
| Southern Sweden | **0.72** | **0.63** | **0.82** |  | **0.66** | **0.46** | **0.95** |  |
| Northern Sweden | **0.59** | **0.49** | **0.70** |  | **0.42** | **0.21** | **0.84** |  |
| Marital status (ref. Married) | **1.34** | **1.18** | **1.51** |  | 0.95 | 0.70 | 1.27 |  |
| Neighborhood deprivation (ref. Low) |  |  |  |  |  |  |  |  |
| Middle | **0.81** | **0.70** | **0.92** |  | 0.87 | 0.59 | 1.27 |  |
| High | 0.82 | 0.67 | 1.00 |  | 0.73 | 0.48 | 1.11 |  |
| Unknown | **0.41** | **0.18** | **0.92** |  | 0.93 | 0.39 | 2.21 |  |
| Diagnosis of diabetes (ref. Non) | 1.15 | 0.96 | 1.39 |  | 1.14 | 0.69 | 1.87 |  |
| Diagnosis of coronary heart disease (ref. Non) | 0.96 | 0.83 | 1.10 |  | 1.09 | 0.71 | 1.67 |  |
| Diagnosis of hypertension (ref. Non) | **1.27** | **1.12** | **1.45** |  | 1.17 | 0.80 | 1.70 |  |
| Diagnosis of stroke (ref. Non) | 1.12 | 0.94 | 1.32 |  | 1.32 | 0.77 | 2.25 |  |
| Diagnosis of depression (ref. Non) | 1.22 | 0.95 | 1.55 |  | 1.05 | 0.63 | 1.72 |  |
| Diagnosis of fracture (ref. Non) | 1.08 | 0.91 | 1.27 |  | 0.95 | 0.54 | 1.68 |  |
| Diagnosis of cancer (ref. Non) | **1.35** | **1.19** | **1.53** |  | 1.30 | 0.89 | 1.90 |  |
| HR: Hazard ratios; CI: confidence interval. | | | | | | | |  |
| *: Full adjusted. |  |  |  |  |  |  |  |  |

Bold values are statistically significant.
